# Epidemiological trends and incidence prediction of periodontal disease based on the Global Burden of Disease study 2021

**DOI:** 10.3389/fdmed.2025.1643049

**Published:** 2025-09-11

**Authors:** Keke Zhang, Yingyi Ma, Jinfang Shi, Yudong Geng

**Affiliations:** ^1^ The Department of Orthodontics, The First Affiliated Hospital of Zhengzhou University, Zhengzhou, China; ^2^ Department of Periodontology, First Affiliated Hospital of Zhengzhou University, Zhengzhou, China; ^3^ Dongyang Traditional Chinese Medicine Hospital, Jinhua, China; ^4^ Department of Oral and Maxillofacial Surgery, The First Affiliated Hospital of Zhengzhou University, Zhengzhou, China

**Keywords:** periodontal disease, disabled life years, long-term trend, global disease burden, ARIMA prediction model

## Abstract

**Objective:**

This study aims to assess the burden and long-term trends of periodontal disease at the global level, and to predict future trends.

**Methods:**

Age-standardized YLD rates (ASYRs) of periodontal disease were obtained from the Global Burden of Disease Study database. Estimated annual percentage change (EAPC) was calculated. The time trend of ASYRs caused by periodontal disease was quantified. ARIMA and ES models were used to predict the future trend of periodontal disease.

**Results:**

In 2021, DALYs and YLDs caused by periodontal disease at the global level were 173.97 person-years and 173.97 person-years, respectively. The number of cases was 5,077,653, the prevalence rate was 27,093/100,000, and the incidence rate was 1,855/100,000. From 1990 to 2021, the burden of periodontal disease showed a downward trend. DALYS, YLDs and prevalence were the highest in low SDI and low-middle SDI areas, but the incidence was lower. In 2021, the incidence rate was the highest in tropical Latin America, and the DALY burden was the heaviest in high-income and low-income areas of the Commonwealth, showing the same trend globally. There was a negative correlation between periodontal disease burden and sociodemographic index (SDI). Based on the ARIMA model, the age-standardized incidence of periodontal disease is expected to decrease to 1,854.07/100,000 by 2050. The age-standardized incidence of periodontal disease will be reduced to 1,858.60/100,000. The ES model is more conservative, and it is expected that by 2050, the age-standardized incidence of periodontal disease will be reduced to 1,828.73/100,000. The age-standardized incidence of periodontal disease will be reduced to 1,841.50/100,000.

**Conclusion:**

ASYRs caused by global periodontal disease have decreased slightly, but they will continue to cause huge losses to healthy life in the future due to population aging and longer life expectancy. It is suggested that the prevention and treatment of periodontal disease should be carried out effectively in combination with the distribution characteristics and causes of periodontal disease in the world.

## 1 Introduction

The global oral health report released by the World Health Organization in 2022 provides a comprehensive picture of the burden of oral diseases for the first time, and emphasizes the need to accelerate the universal coverage of oral health to meet these challenges and opportunities (1). With the increasing prevalence of non-communicable diseases, oral health has become a major public health problem. Periodontal disease refers to the disease that occurs in the supporting tissue of the tooth. It is divided into two categories: gingival disease involving only gingival tissue and periodontitis involving deep periodontal tissue (periodontal ligament, alveolar bone, cementum) (2, 3). Periodontal disease is one of the common oral diseases. The prevalence of periodontal disease in developing countries remains high and increases with age (4, 5). Moreover, periodontal disease is also a major oral disease that endangers human health (6). Studies have confirmed that periodontal disease is closely related to diabetes, chronic kidney disease, cardiovascular disease and other systemic diseases (7–9). A two-year longitudinal study revealed that patients with diabetes and severe periodontitis had a six-fold increased risk of worsened glycemic control compared to diabetic patients without periodontitis (10). A meta-analysis investigating the association between periodontal disease and cardiovascular events demonstrated that subjects with periodontal disease exhibited significantly higher odds and elevated risk of developing cardiovascular diseases (11). A Chinese study evaluating oral health-related quality of life in type 2 diabetes patients with chronic periodontitis revealed that periodontal disease significantly impairs masticatory function, verbal communication, and social interaction, thereby reducing overall quality of life. These findings underscore the substantial burden of periodontal disease (12), suggesting that the burden of periodontal disease is serious.

In order to clarify the burden of periodontal disease, this study describes and analyzes the global burden of periodontal disease based on the 2021 Global Burden of Disease (GBD) database, aiming to provide a reference for the formulation of prevention and treatment strategies for periodontal disease, so as to reduce the burden of periodontal disease and ensure global oral health. Therefore, based on the data of the Global Burden of Disease Database in 2021, this study counted the incidence and mortality of global periodontal disease, estimated its disease burden and development trend in different countries or regions, and aimed to provide an objective basis for formulating effective prevention and control strategies for periodontal disease and reducing the global disease burden.

## 2 Materials and methods

### 2.1 Data source

The data of this study are from the global burden of disease research database. The database provides a comprehensive and systematic assessment of the global burden of 369 diseases, injuries and injuries and 87 risk factors in 204 countries and territories between 1990 and 2021. This study extracted global, regional and national periodontal disease morbidity, mortality and disability-adjusted life year data from the database.

### 2.2 Research indicators and definitions

In GBD2021, periodontal disease is defined as: community index of periodontal treatment needs (CPITN) reaches grade IV, attachment loss (AL) > 6 mm, or pocket depth (PD) > 5 mm. The definition of periodontal disease is based on the International Classification of Diseases, tenth revised edition (ICD10), coded as K05K06.9; the code of ICD9 is 523,523.9. Socio-demographic index (SDI) is a comprehensive index to measure the development level of a region or a country based on three dimensions: per capita income of lagging distribution, average education level of population aged 15 and above, and total fertility rate of population under 25 years old. The SDI value ranges from 0 to 1, and the larger the value, the higher the socio-economic development level of the region or country. IHME evaluated the SDI level of each country, and we obtained the SDI values of 204 countries from 1990 to 2021 from the GBD official website. Based on SDI values, 204 countries and territories are divided into five regions: low SDI (0.81) regions.

### 2.3 Statistical analysis

DALY and YLDs are comprehensive time-based measures of the number and quality of life, and are currently recognized as one of the most useful global burden of disease indicators. In this study, the disease burden was quantified by calculating the incidence, mortality and DALY of periodontal disease. The specific method has been elaborated in the study of GBD collaborators (13). In order to ensure the comparability of statistical indicators, age-standardized rates (ASRs) were used to reduce the impact of changes in population structure and age distribution over time. The trends of age-standardized incidence rate (ASIR), age-standardize prevalence rate (ASPR) and age-standardized DALY rate (ASDR) were evaluated by estimated annual percentage change. The 95% confidence interval (95% CI) of EAPC was calculated by linear regression model. If the lower limit of 95% CI of EAPC was greater than 0, it was considered that ASRs increased during the whole observation period. If the upper limit of 95% CI of EAPC was less than 0, it was considered that ASRs decreased. Cluster analysis was performed using the K-means algorithm to partition the samples, with Euclidean distance employed as the metric for measuring similarity. The kmeans function from the stats package was utilized, and the computation was repeated 10 times to reduce the random bias caused by initial cluster centers. The calculations were iterated continuously until convergence was achieved (14). The data were analyzed and plotted by R4.2.3.

### 2.4 Prediction models

In order to understand the future of the global burden of periodontal disease, this study used time series analysis to predict the burden of periodontal disease from 1990 to 2021 to 2050 using known data.

Auto regressive Integrated Moving Average model (ARIMA) is one of the most commonly used time series analysis methods. It has high prediction accuracy and is widely used in various fields related to time units. It is a hybrid model composed of autoregressive model (AR) and moving average model (MA), so it is also called integrated average autoregressive model (15). The model expressions are ARIMA (p, d, q).

Exponential Smoothing (ES) (16) is one of the most commonly used prediction methods in time series analysis. It is suitable for short-term prediction, especially for trend or seasonal data. The core idea is to assign an exponential decreasing weight to historical data (the weight of recent data is higher, and the weight of long-term data is lower), so as to highlight the impact of recent data.

## 3 Results

### 3.1 The burden of periodontal disease at the global level

**Figure 1:**
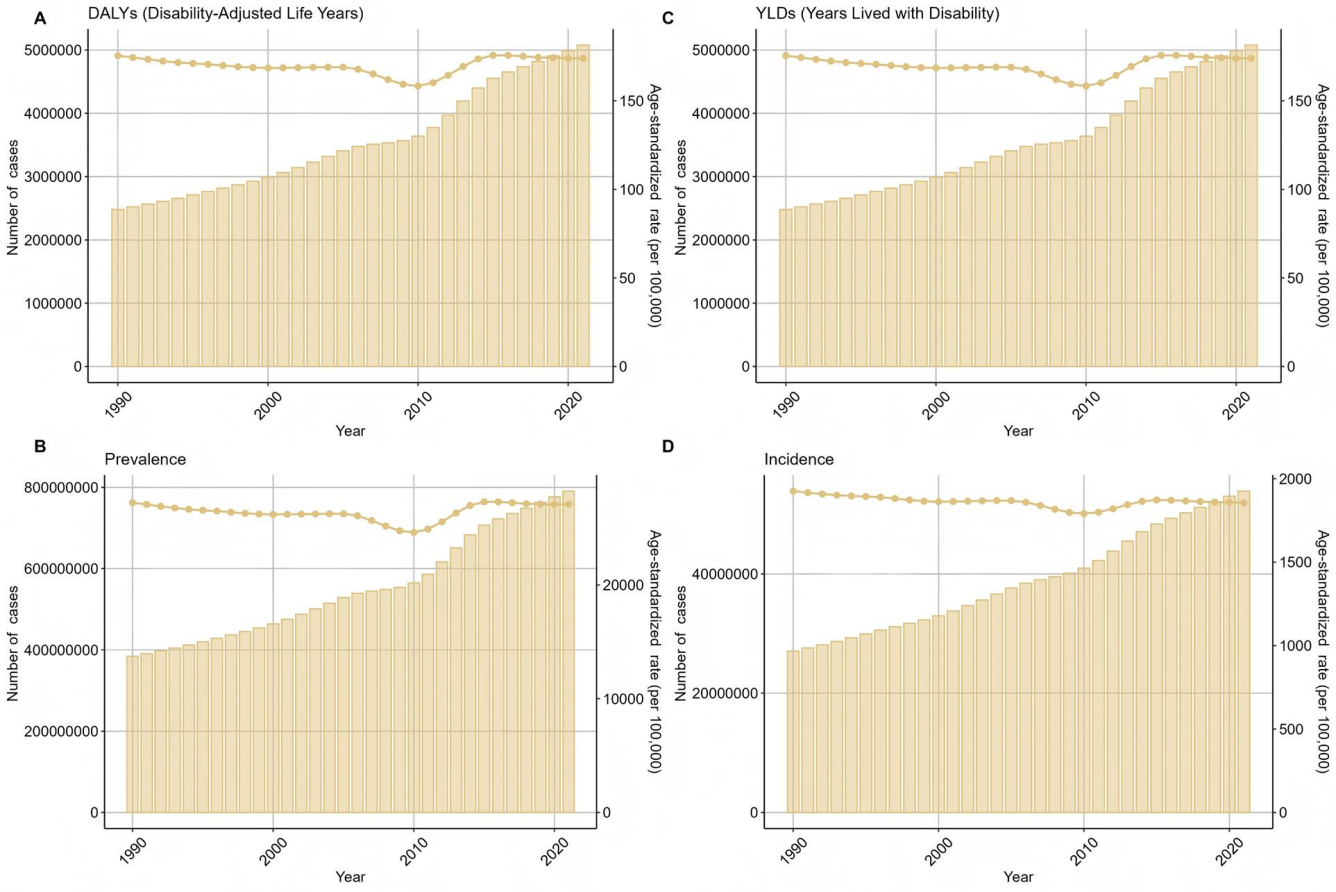
The overall trend of the number and standardized rate of periodontal disease in the world from 1990 to 2021 **(A)** DALYs **(B)** YLDs **(C)** prevalence **(D)** incidence.

We analyzed the long-term trend of global periodontal disease from 1990 to 2021, and the results are shown in Figure 1. Compared with 1990, the incidence of periodontal disease in the world increased by 99.22%. In 2021, the DALYs caused by periodontal disease at the global level was 173.97 person-years, the number of cases was 5,077,653, the YLDs was 173.97 person-years, the number of cases was 5,077,653, the prevalence rate was 27,093/100,000, the number of cases was 790,657,510, the incidence rate was 1,855/100,000, and the number of cases was 53,893,262. From 1990 to 2021, the disease burden of periodontal disease showed a downward trend, with the highest incidence rate of 1,925/100,000 in 1990 (Figure 1D) and the lowest incidence rate of 1,790/100,000 in 2010. The global incidence of periodontal disease showed an overall downward trend from 1990 to 2010, and the incidence rate showed a slight upward trend since 2010.

**Figure 2:**
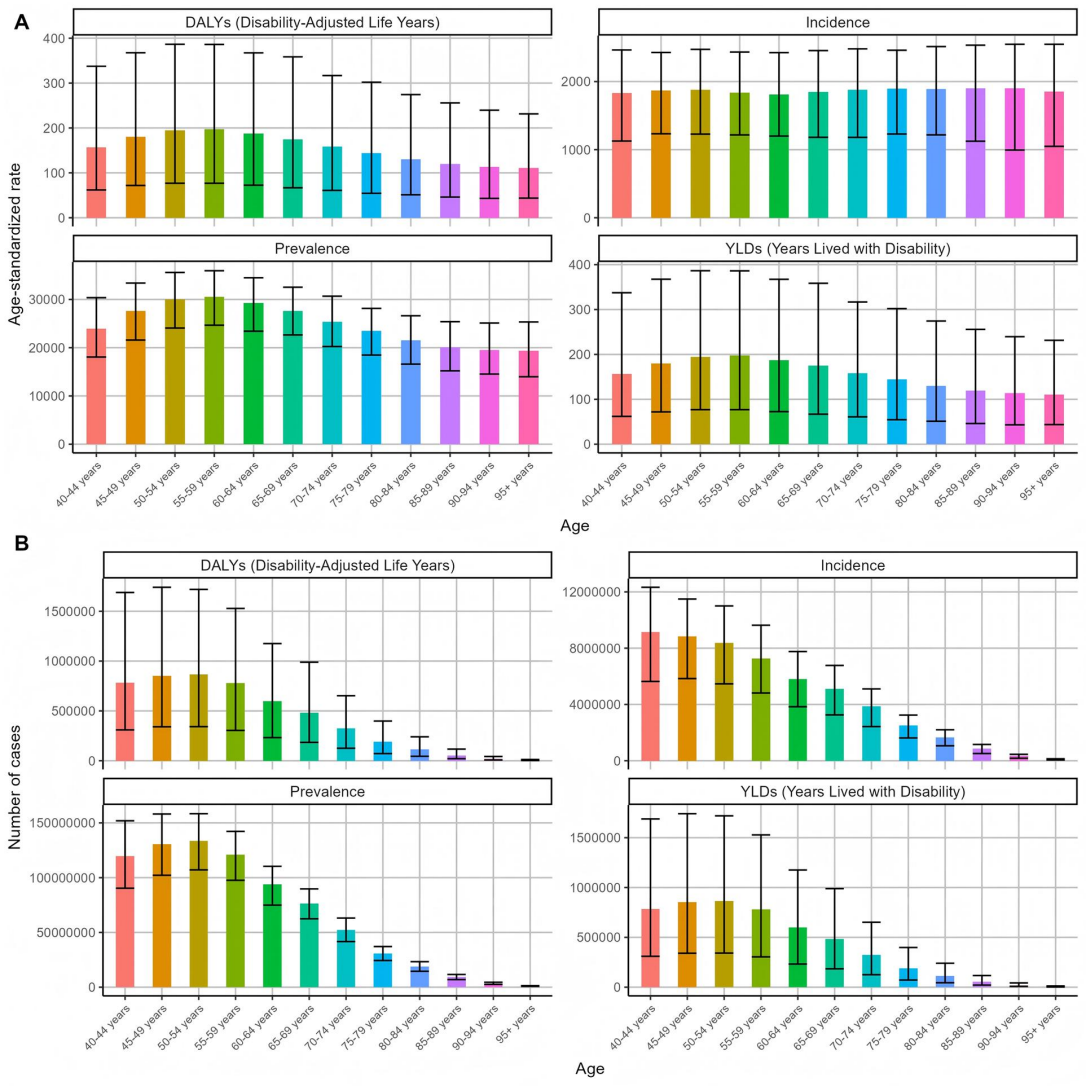
Global distribution of periodontal disease burden in different age groups in 2021 **(A)** Age-standardized rate **(B)** number of cases.

The global burden of periodontal disease in 2021 is unevenly distributed among all age groups. The overall number and prevalence of periodontal disease increase first and then decrease with age. The distribution of periodontal disease in 40–54 years old showed a continuous and rapid upward trend, and the growth rate increased rapidly with age. The number of patients increased the fastest in the 45–49 age group, and the prevalence rate increased the fastest in the 40–44 age group. The specific prevalence distribution is shown in Figure 2. The number of new cases of periodontal disease in 2021 gradually decreased with age.

**Figure 3:**
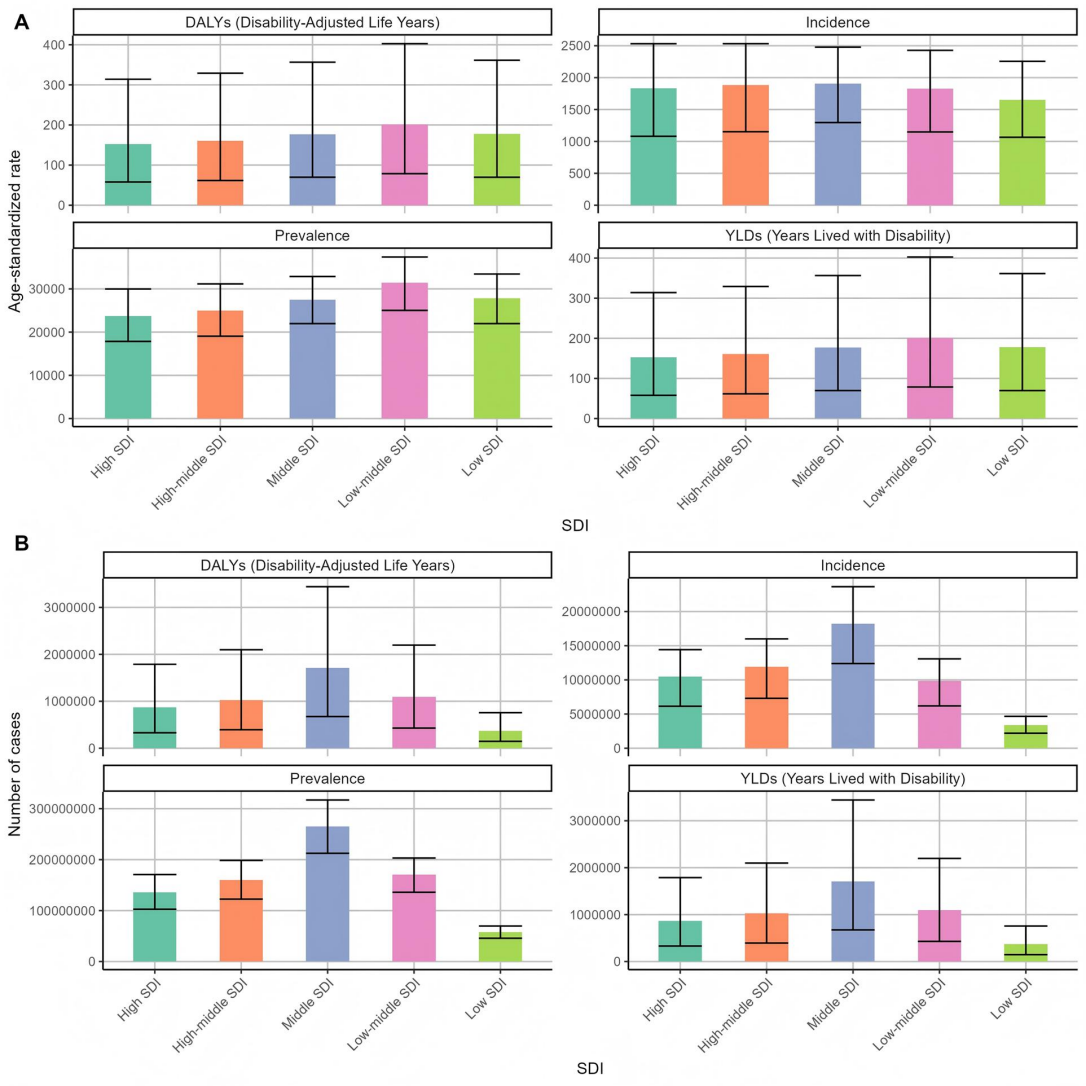
Distribution of periodontal disease burden of different SDIs in the world in 2021 **(A)** Age-standardized rate **(B)** number of cases.

**Figure 4:**
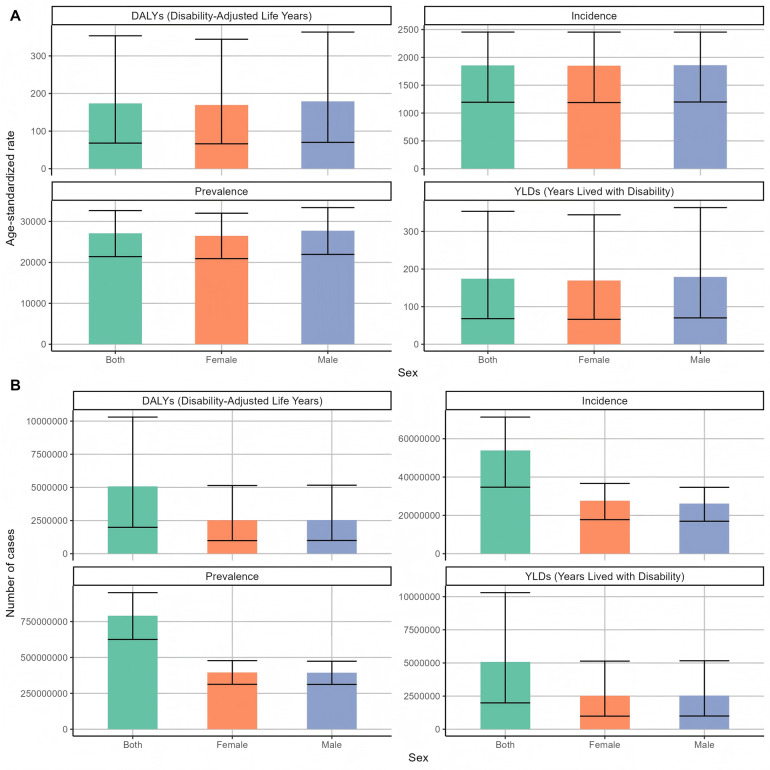
Global gender-specific periodontal disease burden distribution in 2021 **(A)** Age-standardized rate **(B)** number of cases.

**Figure 5:**
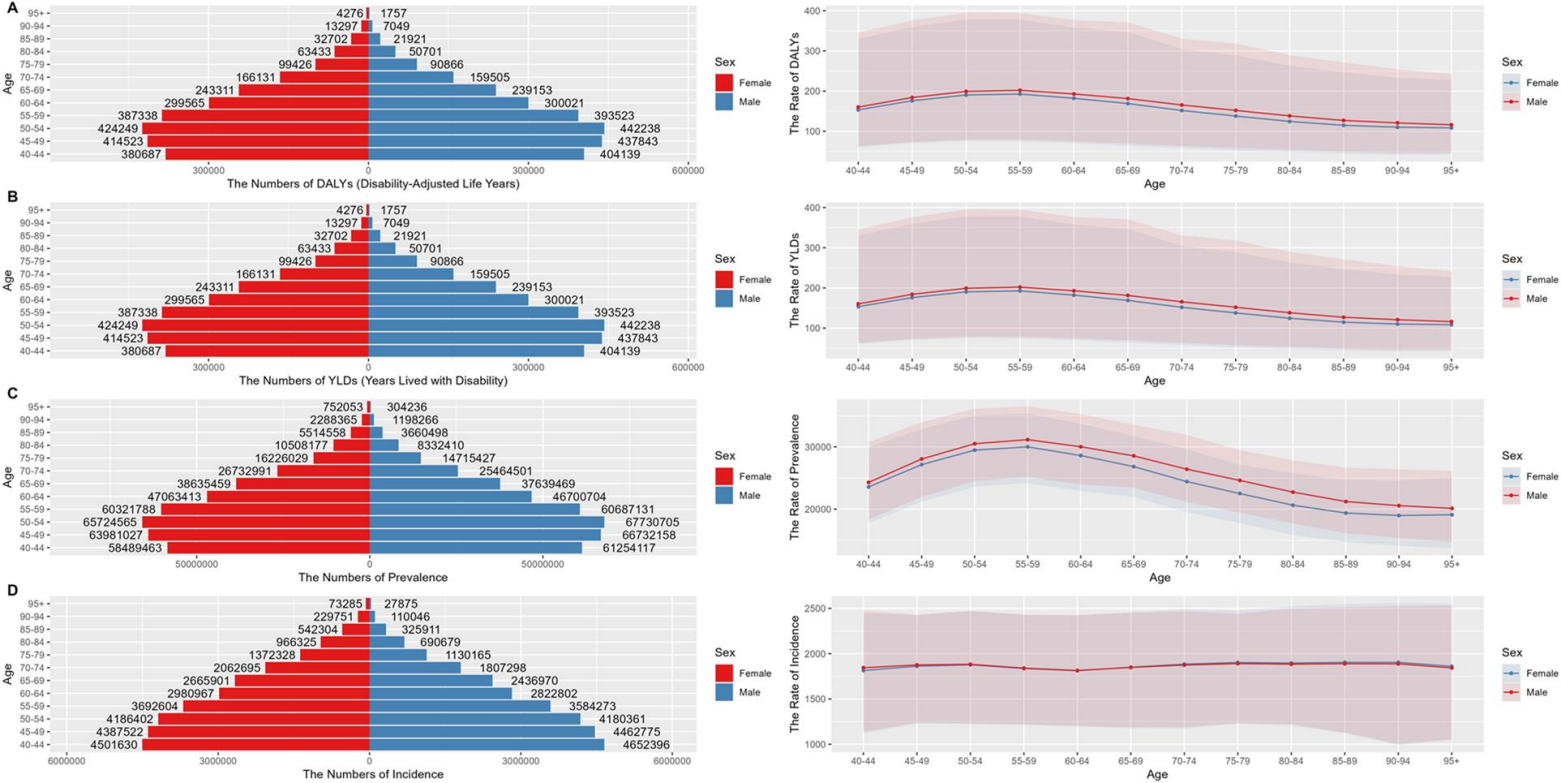
Distribution of periodontal disease burden in men and women of different ages in the world in 2021 **(A)** DALYs **(B)** YLDs **(C)** prevalence **(D)** incidence.

By comparing the disease burden of different SDI levels in 2021 (Figure 3), it was found that the ASYRs in the areas with low and medium SDI levels were the highest, 200.97 person-years; the ASYRs with high-SDI levels were the lowest at 152.37 person-years. The results of DALYs index of periodontal disease burden of different genders in 2021 (Figure 4) showed that the DALYs caused by periodontal disease to men and women were 179.05 and 169.26 person-years, respectively, and the standardized incidence rates were 1,858.78/100,000 and 1,851.64/100,000, respectively. The overall DALYs index is unevenly distributed among men and women of all ages in China (Figure 5). The DALYs caused by periodontal disease increased with age, reaching a peak in the 50–54 age group (424,249 and 442,238). After that, DALYs gradually decreased. The standardized DALY rate showed a similar trend, with a peak in the 55–59 age group (197.32 person-years).

**Figure 6:**
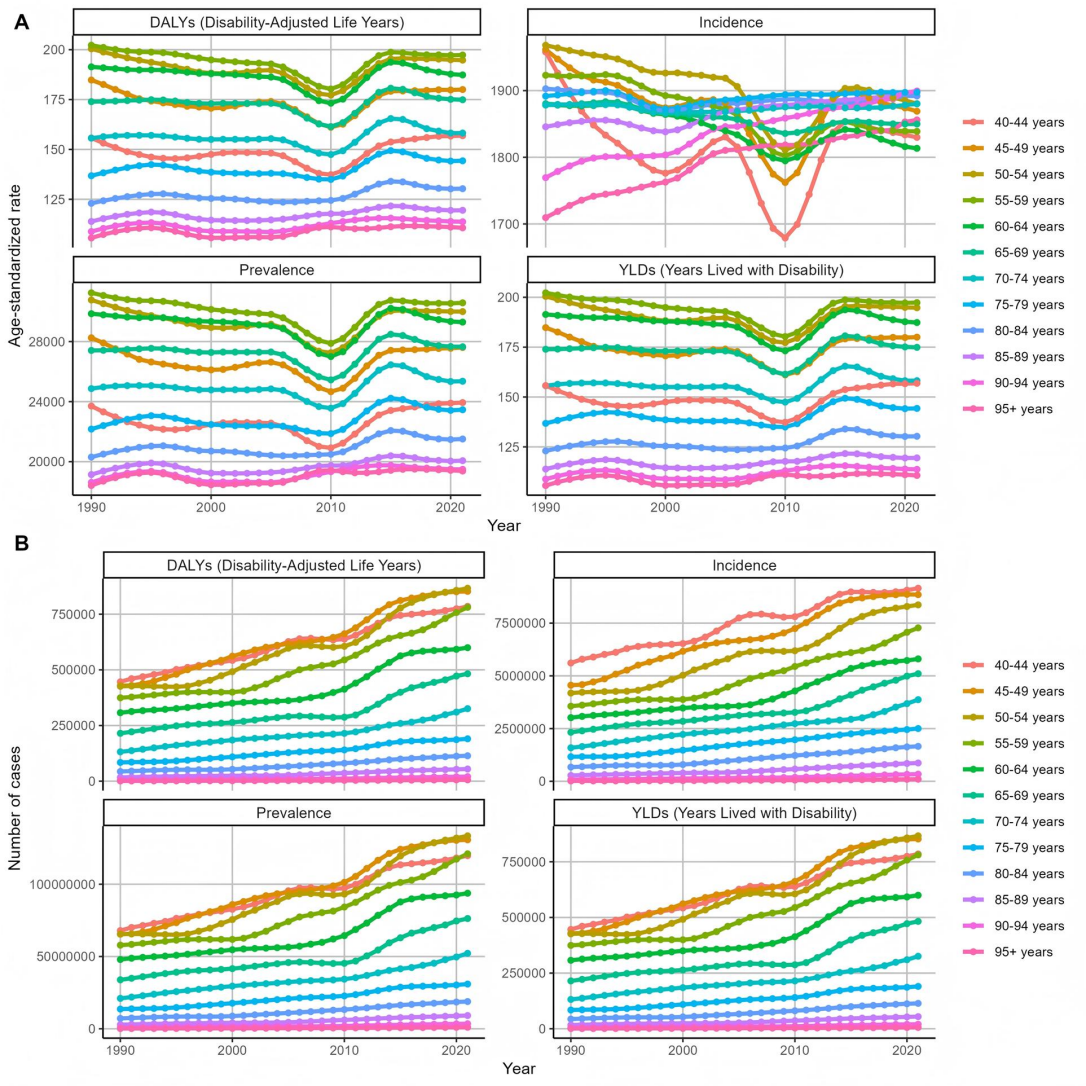
Trends in the distribution of periodontal disease burden in different age groups in the world from 1990 to 2021 **(A)** Age-standardized rate **(B)** number of cases.

**Figure 7:**
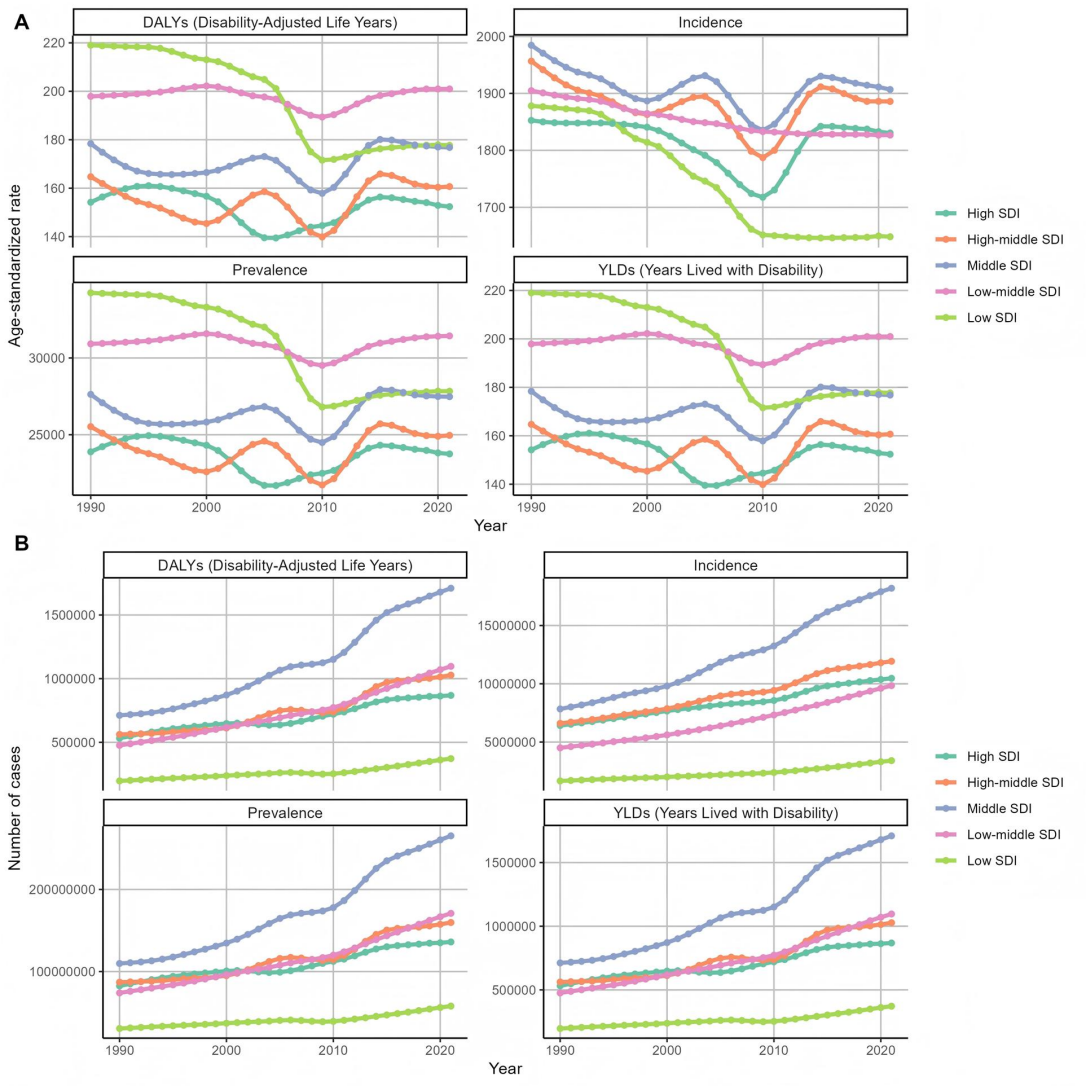
Trends in the distribution of periodontal disease burden of different SDIs in the world from 1990 to 2021 **(A)** Age-standardized rate **(B)** number of cases.

**Figure 8:**
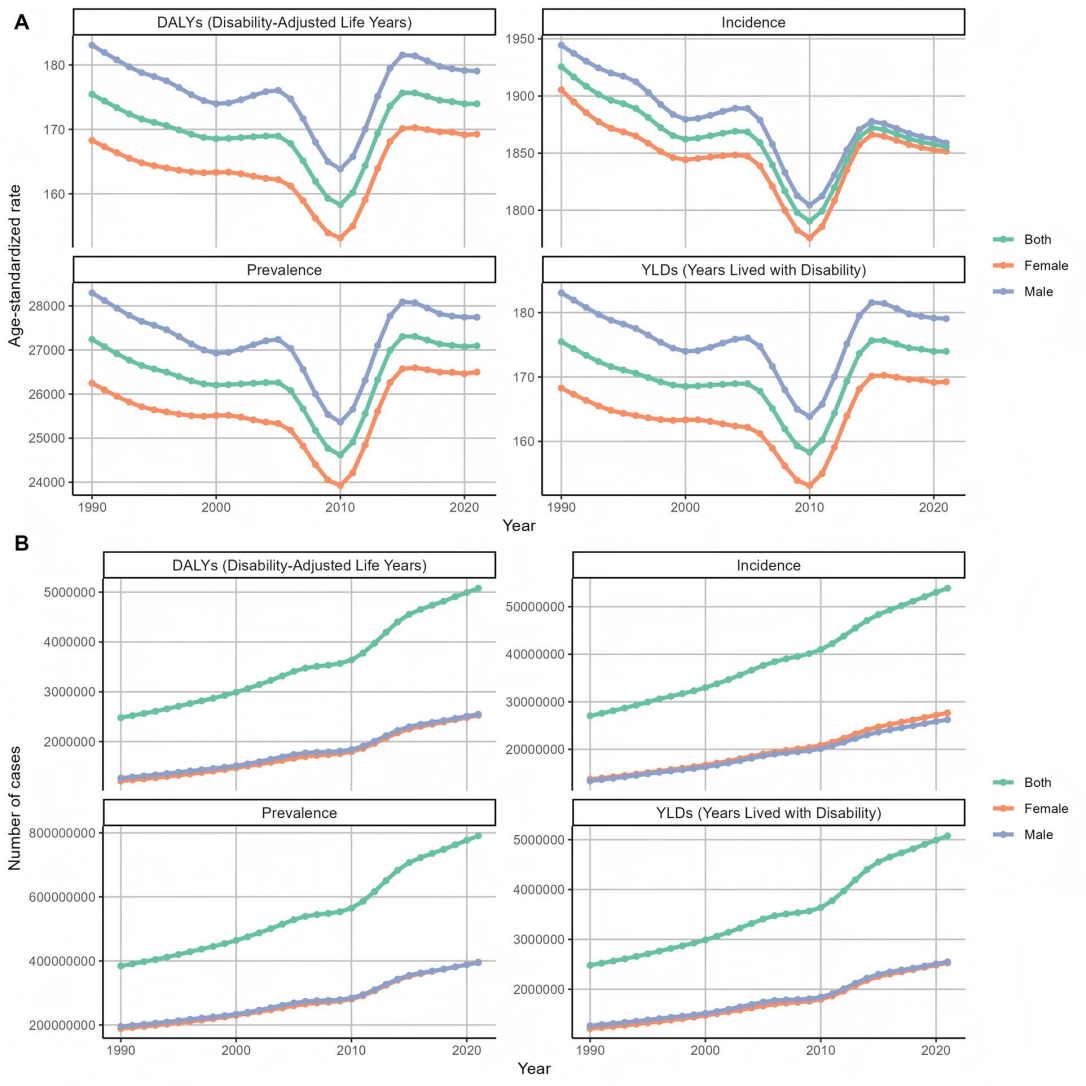
Trends in the distribution of periodontal disease burden in different genders in the world from 1990 to 2021 **(A)** Age-standardized rate **(B)** number of cases.

**Figure 9:**
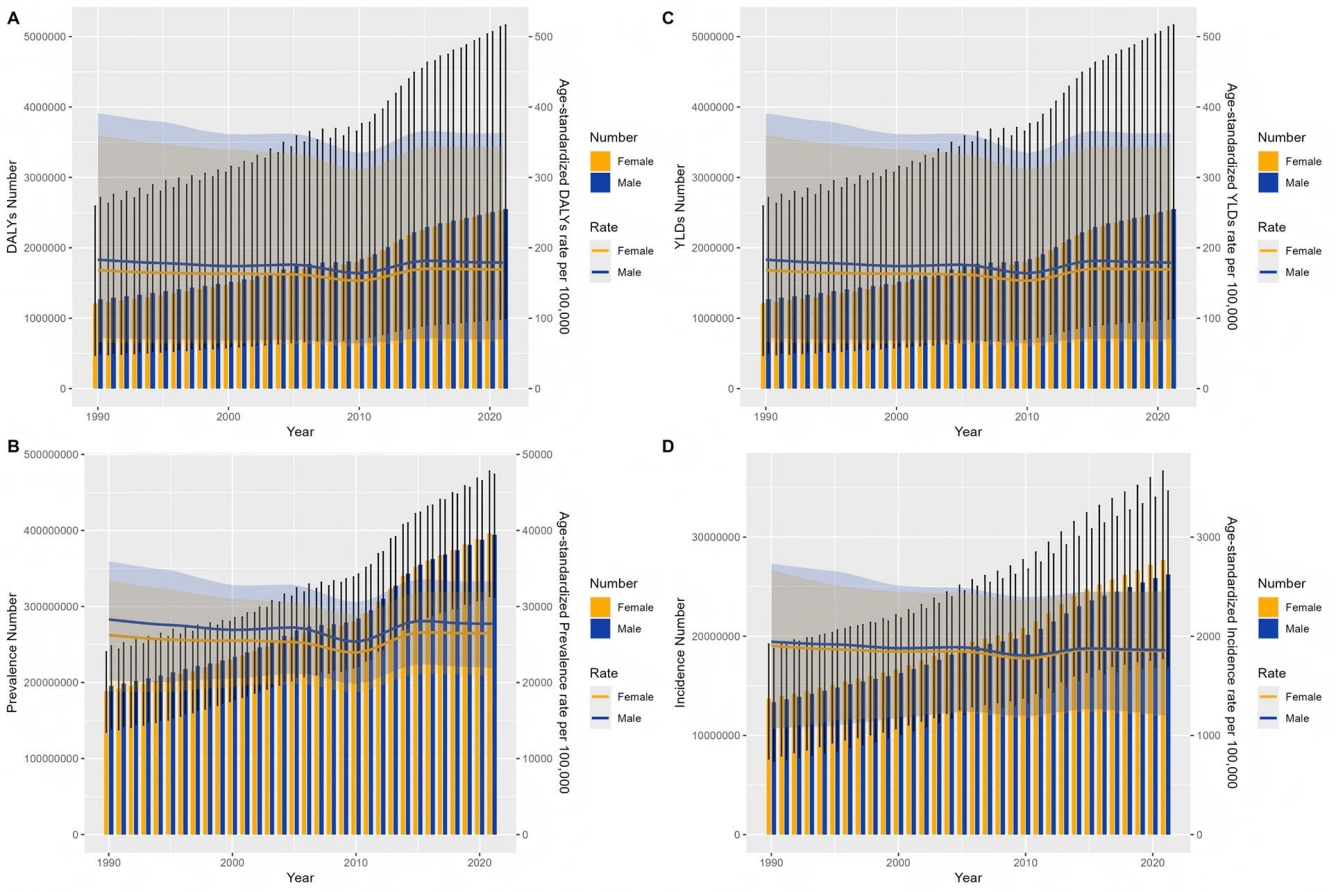
Global trends in the distribution of periodontal disease burden in men and women from 1990 to 2021 **(A)** DALYs **(B)** YLDs **(C)** prevalence **(D)** incidence.

After that, we classified and analyzed the global burden of periodontal disease from 1990 to 2021 in detail. The trend of the burden of disease in all age groups was basically the same (Figure 6), which was consistent with the above slow decline. The burden was the lowest in 2010, and the growth trend of the number of cases, whether DALY or YLDs, was slow growth. Then we analyzed the trend of disease burden in areas with different SDI levels (Figure 7). The DALYS, YLDs and prevalence rates in low SDI and low-medium SDI areas were the highest, but the incidence rate was at a lower position. The number of cases in the medium SDI area was the highest in both incidence rate, prevalence rate and DALYS, and the upward trend was obvious. From 1990 to 2021, the global standardized YLD rate of periodontal disease by gender showed a downward and upward trend. The incidence of male patients was higher than that of female patients, and the downward trend was lower than that of female patients. Among them, from 1990 to the end of the last century, the standardized YLD rate of periodontal disease in both sexes remained basically stable; from the beginning of this century to 2010, the global standardized YLD rate of periodontal disease in all genders showed a significant downward trend; from 2010 to 2015, the global standardized YLD rate of periodontal disease showed an upward trend in both men and women. After 2015, the trend tended to be stable. The trend of standardized YLD rate of periodontal disease in the global population from 1990 to 2021 is shown in Figure 8. Figure 9 is a more intuitive description of the trend of global periodontal disease burden distribution in men and women.

### 3.2 The burden of periodontal disease at the regional level

**Figure 10:**
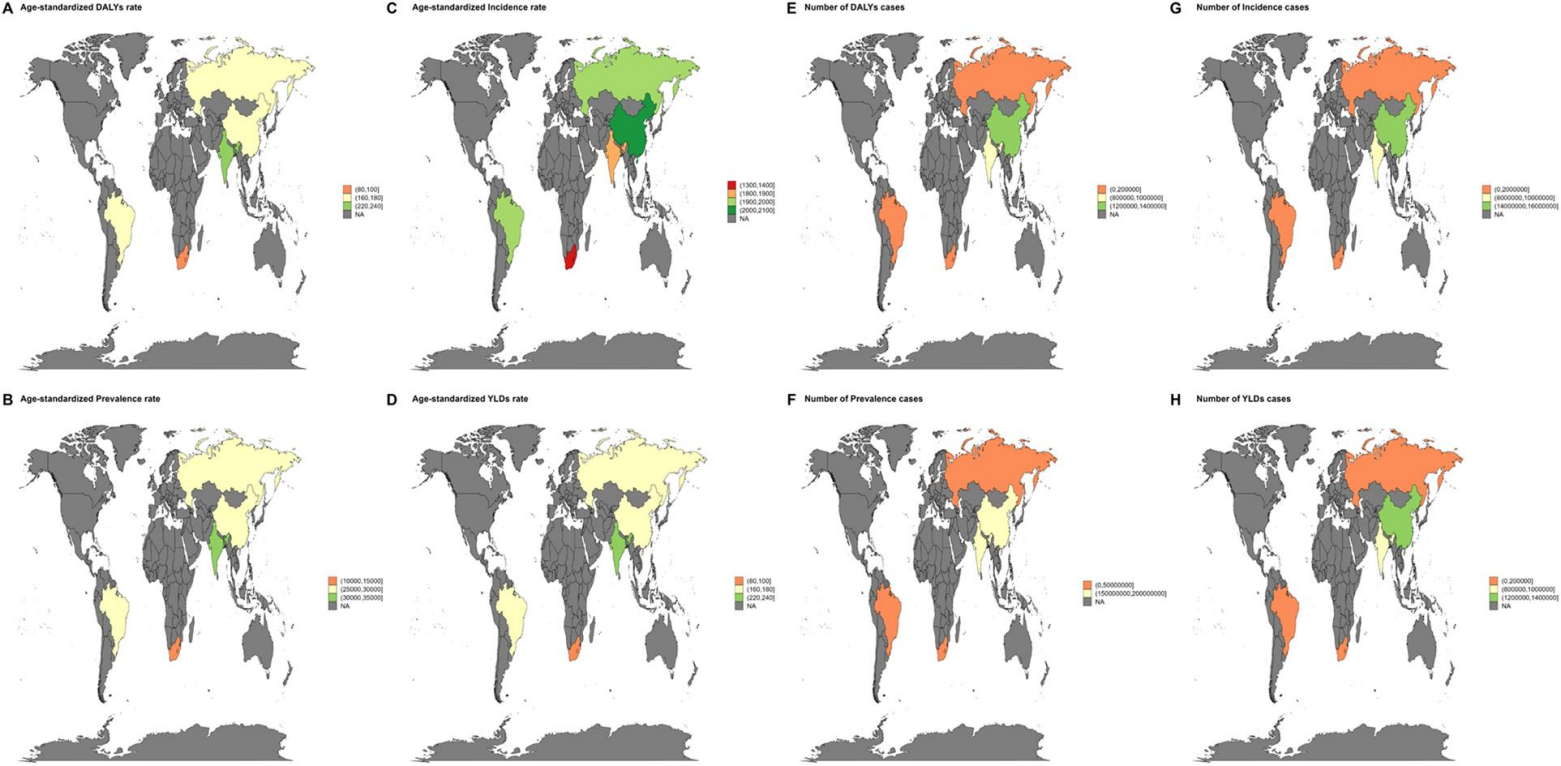
Global burden of periodontal disease. **(A–D)** Standardized rate burden due to periodontal disease in 2021 (DALYs, YLDs, prevalence, incidence) **(E–H)** The number of cases due to periodontal disease in 2021 (DALYs, YLDs, prevalence, incidence).

The distribution of periodontal disease burden in countries or regions is shown in Figure 10. In 2021, the top three countries in the burden of periodontal disease were China (2,003.9/100,000), Russia (1,997.4/100,000), and the United Kingdom (1,982.2/100,000). The corresponding DALYs were 172.9 person-years in China, 164.5 person-years in Russia, and 168.9 person-years in the United Kingdom. In 2021, the geographical area with the largest number of patients with periodontal disease is China. Whether it is from the age standardization rate or the number of cases, the disease burden of periodontal disease in China is high.

**Figure 11:**
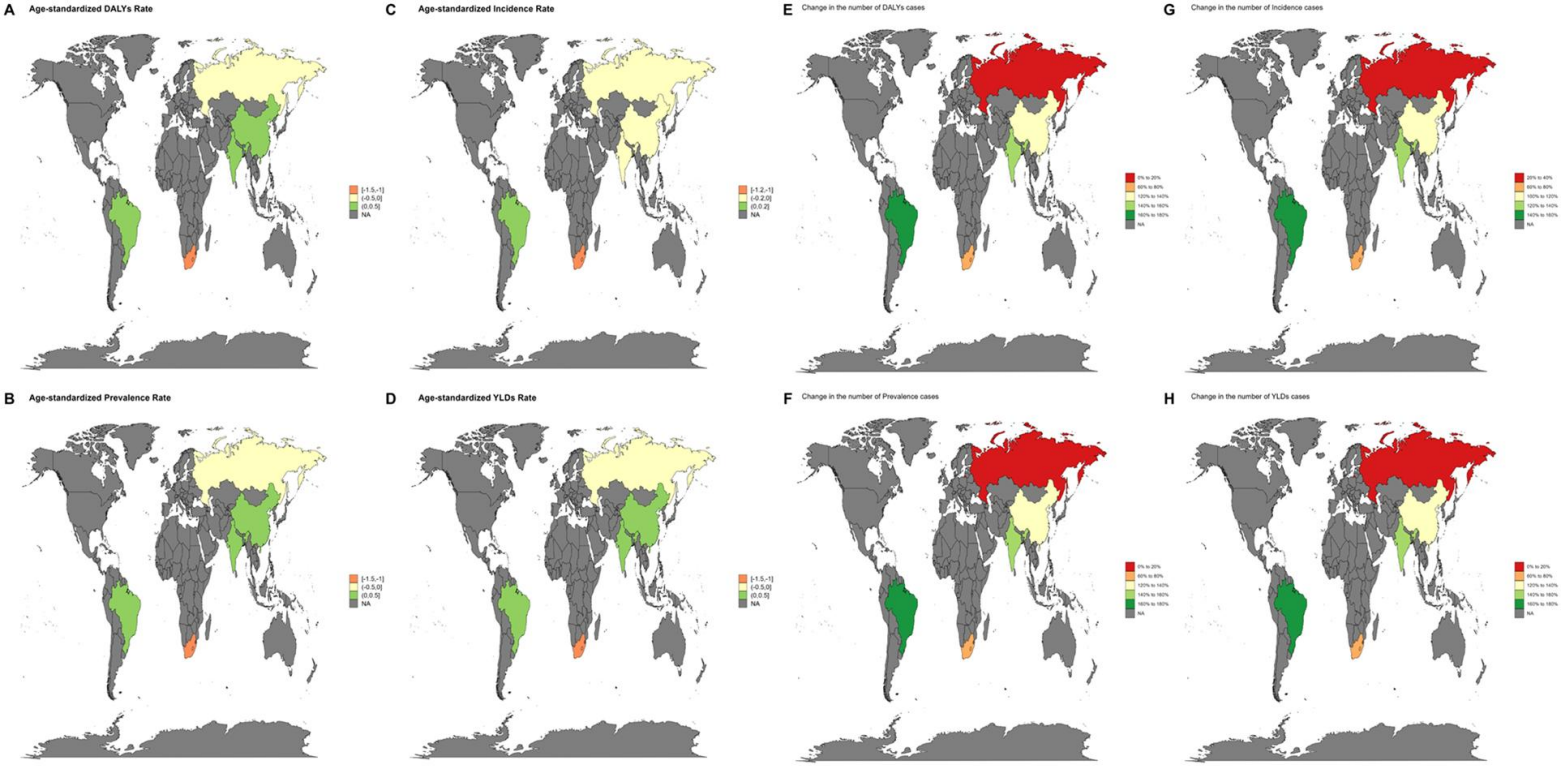
Trends in the global burden of disease due to periodontal disease from 1990 to 2021 **(A–D)** changes in the EAPC burden due to periodontal disease from 1990 to 2021 (DALYs, YLDs, prevalence, incidence) **(E–H)** percentage change in the number of cases due to periodontal disease in 2021 (DALYs, YLDs, prevalence, incidence).

From 1990 to 2021, ASYRs in most countries and regions showed a decreasing trend, and the burden of periodontal disease in the United Kingdom (EAPC = 0.44,95% UI: 0.20–068) and China (EAPC = 0.34,95% UI: 0.03–0.66) showed an increasing trend (Figure 11). The country with the largest increase in the number of cases is South Africa, and Russia's long-term growth rate is relatively flat.

**Figure 12:**
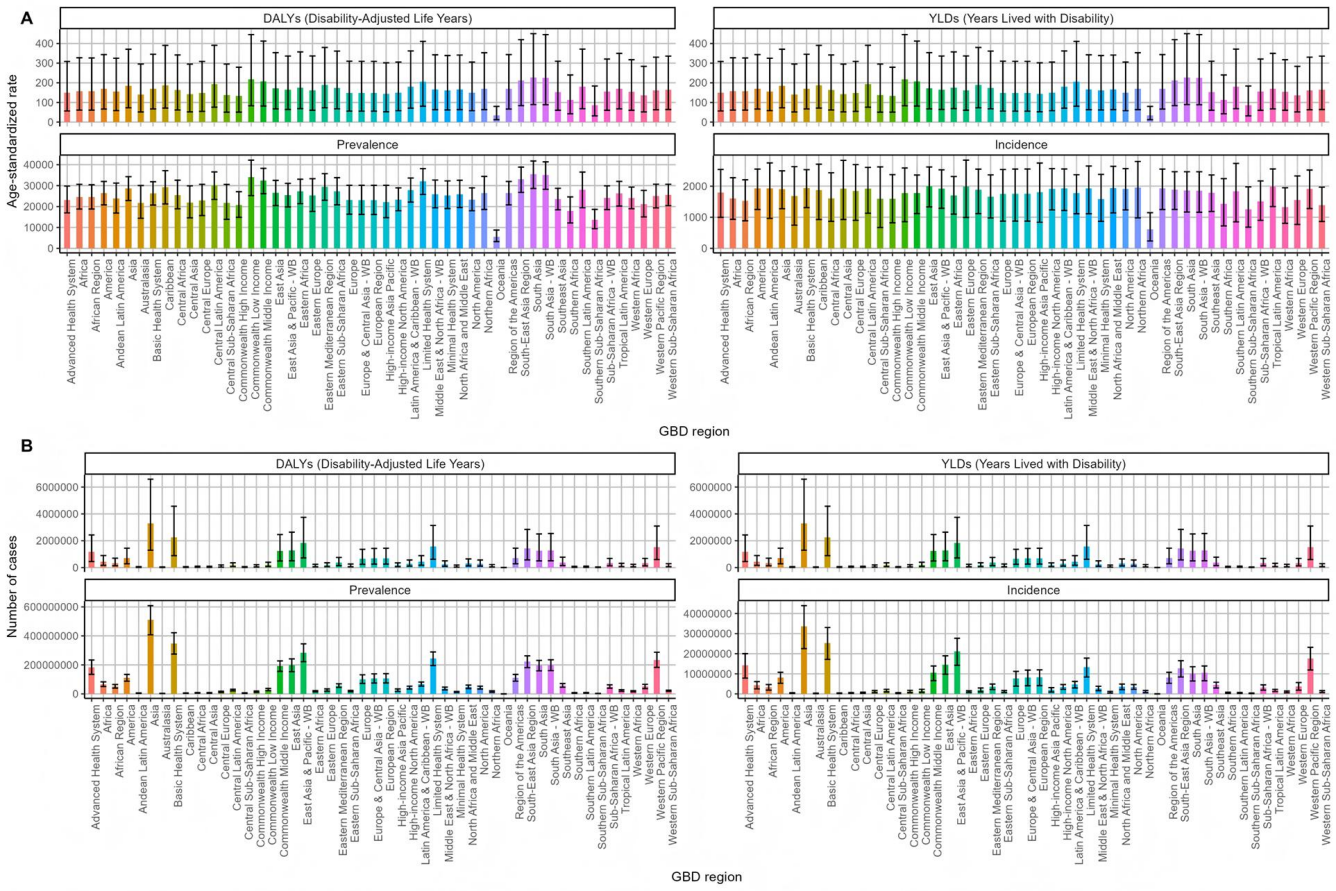
Disease burden caused by periodontal disease in the world. **(A)** Age-standardized rate **(B)** Number of cases.

**Figure 13:**
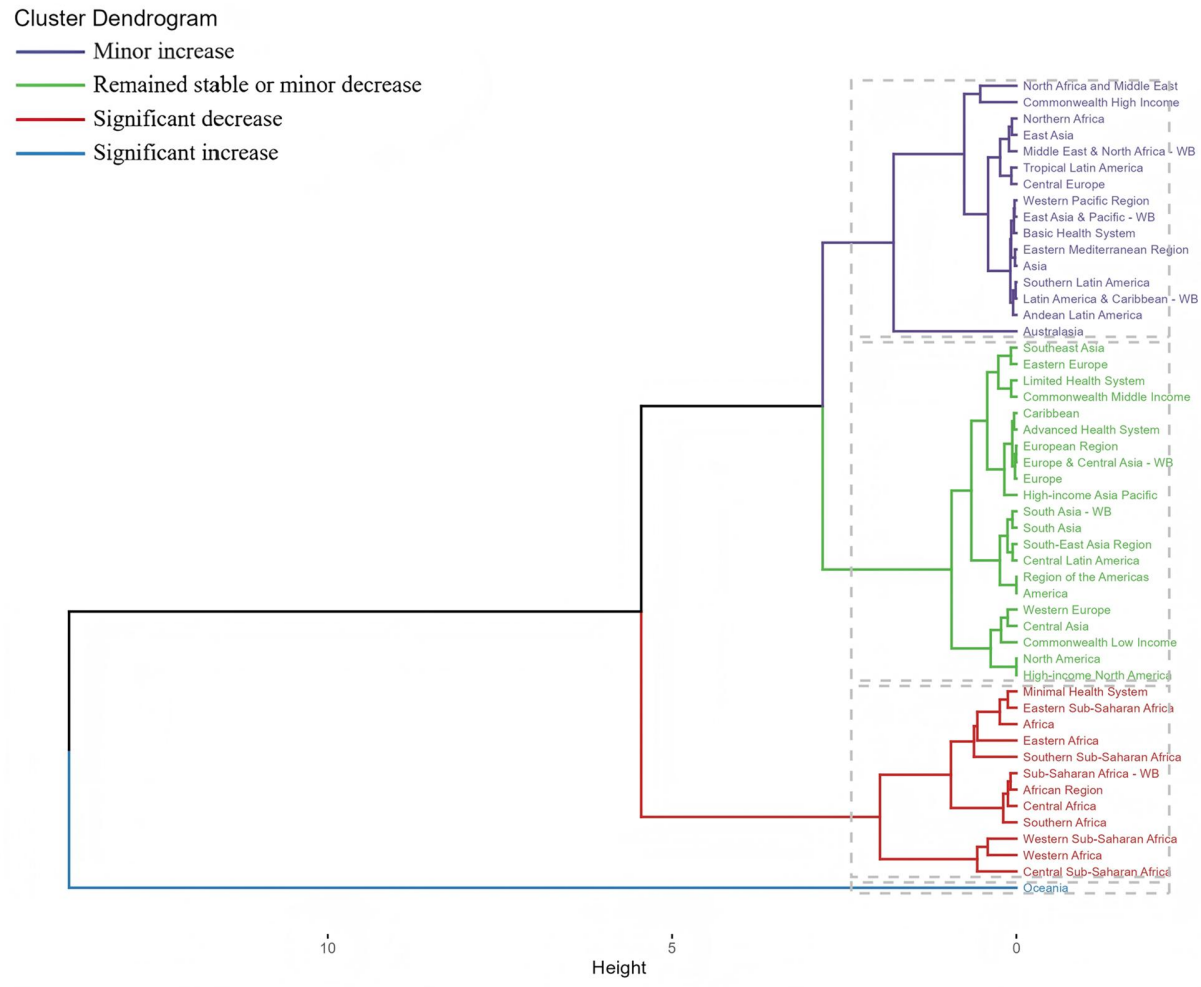
National clustering of changes in the global disease burden due to periodontal disease from 1990 to 2021.

In 2021, the incidence of tropical Latin America was the highest, and the DALY burden in the high-income and low-income areas of the Commonwealth was the heaviest. The global trend was the same (Figure 12). In terms of the number of cases, Latin America and Australia had the highest prevalence and incidence. In contrast, Central Europe, high-income North America and Central Asia have less burden. The cluster analysis tree diagram showed that the countries with a slight increase in the disease burden were the Middle East, Central Europe, Asia and Latin America; slightly reduced are the Americas, Europe and high-income Asia-Pacific region; the significant decrease is mainly in the African region, which may be caused by the rapid improvement of medical level (Figure 13).

**Figure 14:**
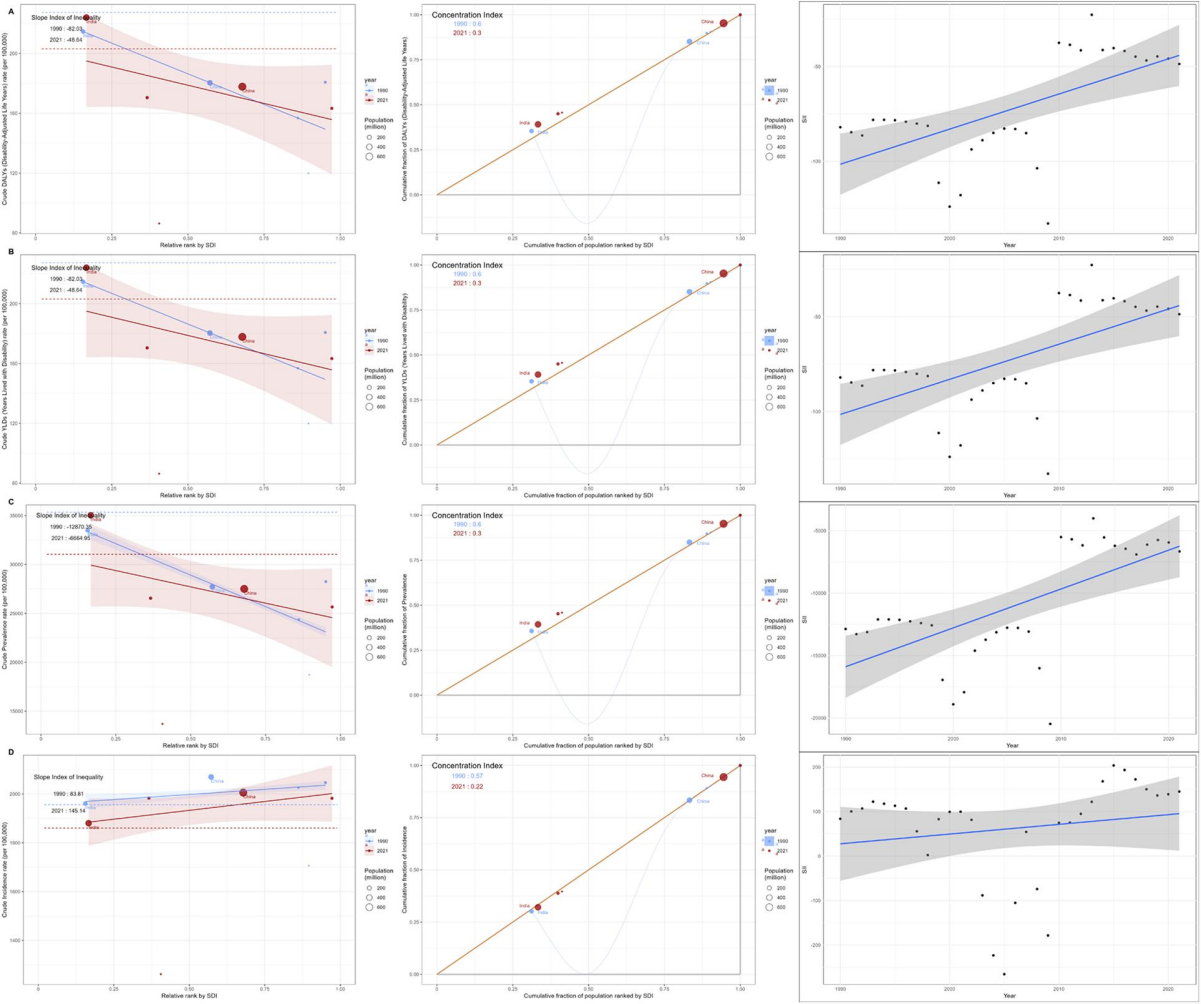
Global periodontal health status **(A)** DALYs **(B)** YLDs **(C)** prevalence **(D)** incidence, 1990–2021.

In 1990–2021, the global ASYR, ASPR and ASDR slope index decreased with the increase of SDI, and the ASIR slope index increased with the increase of SDI, while the concentration index decreased significantly in the same period. In the analysis of absolute health inequality, the ASDR slope index increased from−82.03 in 1990 to−48.64 in 2021, that is, the difference in health outcomes among people with different socioeconomic status is decreasing. The slope index of ASPR and ASYR showed a downward trend, while ASIR showed an upward trend. In the relative health inequality analysis, the ASIR, ASYR, ASPR and ASDR concentration index decreased significantly from 1990 to 2021, which also verified the previous results that health inequality was no longer so serious (Figure 14).

### 3.3 Prediction of global future periodontal disease burden

**Figure 15:**
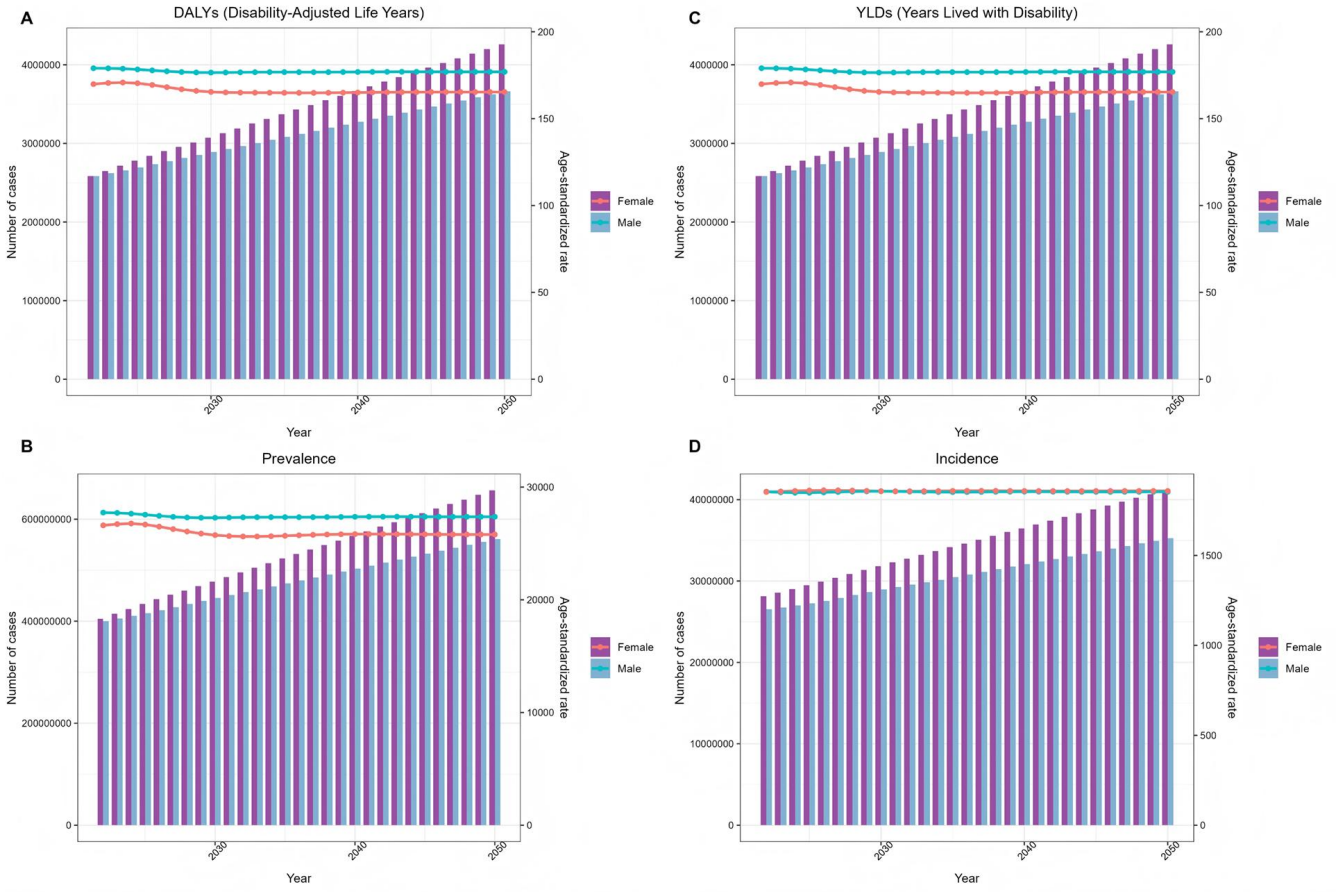
ARIMA model predicts changes in the global burden of periodontal disease from 2022 to 2050 **(A)** DALYs **(B)** prevalence **(C)** YLDs **(D)** incidence.

Based on the ARIMA model, this study predicts the value of periodontal disease-related disease burden in China from 2022 to 2050, and the results are shown in Figure 15. The future burden of periodontal disease in China will show a slight increase and then a downward trend. After 10 years, the burden of periodontal disease in China will be reduced. It is estimated that by 2050, the age-standardized incidence of periodontal disease in men will be reduced to 1,854.07/100,000. The age-standardized incidence of periodontal disease in men will be reduced to 1,858.60/100,000.

**Figure 16:**
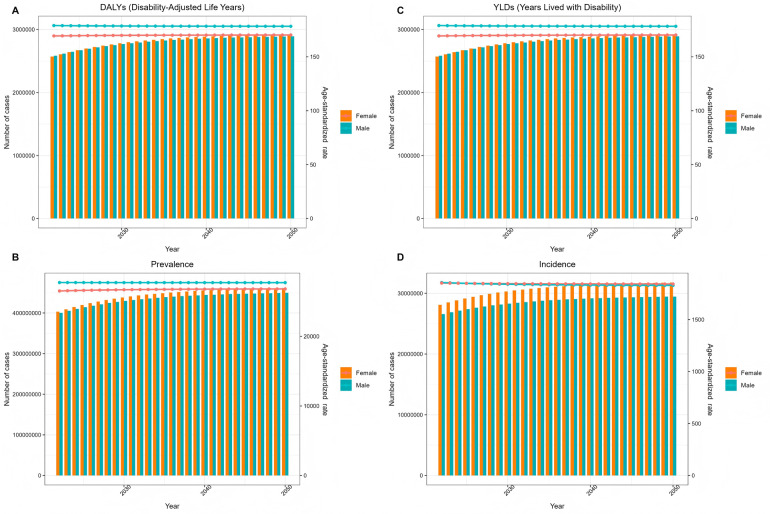
Es model predicts changes in the global burden of periodontal disease from 2022 to 2050 **(A)** DALYs **(B)** prevalence **(C)** YLDs **(D)** incidence.

At the same time, the time series data calculated by ES model based on exponential weighted average are more conservative than ARIMA model. The results are shown in Figure 16, and the fluctuation range of the curve is smaller. It is expected that by 2050, the age-standardized incidence of male periodontal disease will be reduced to 1,828.73/100,000. The age-standardized incidence of periodontal disease in men will be reduced to 1,841.50/100,000.

## 4 Discussion

Consistent with previous research findings (17), this study conducted a systematic analysis of the global disease burden and long-term trends of periodontitis based on the 2021 Global Burden of Disease (GBD) data, utilizing both ARIMA and ES models to predict future trends. The results demonstrate that although the age-standardized rates (ASYRs) of periodontitis show a declining trend globally, its disease burden remains significant, particularly in low and middle-low SDI regions.

This study found that in 2021, periodontitis caused 173.97 disability-adjusted life years (DALYs) globally, with a prevalence rate of 27,093 per 100,000 population and an incidence rate of 1,855 per 100,000 population. From 1990 to 2021, the global burden of periodontitis generally showed a declining trend, although a slight increase in incidence was observed after 2010. This trend may be associated with factors such as population aging, improved oral health awareness, and uneven distribution of medical resources (18). Notably, low and middle-low SDI regions bore the heaviest disease burden, while high SDI regions had a relatively lighter burden, indicating a strong correlation between socioeconomic status and the incidence and burden of periodontitis (19).

The burden of periodontal disease exhibits a negative correlation with the Socio-demographic Index (SDI), with low-SDI regions demonstrating significantly higher DALYs, YLDs, and prevalence rates compared to high-SDI areas. These findings suggest that regions with lower socioeconomic development may experience elevated periodontal disease prevalence and burden due to inadequate oral healthcare resources, insufficient health awareness, and ineffective preventive measures (18). This phenomenon may be closely associated with the following socioeconomic factors:

1. Unequal distribution of healthcare resources

Low-SDI regions often face a scarcity of oral healthcare resources, including low dentist-to-population ratios, insufficient specialized periodontal treatment equipment, and limited coverage of preventive oral health programs. For instance, in Tropical Latin America and certain African regions, the prevalence of periodontal disease is notably high, yet the accessibility of oral health services remains significantly lower than in high-SDI countries (e.g., Western Europe and North America) (20). Such disparities in resource allocation hinder early intervention for periodontal disease, thereby exacerbating its disease burden.

2. Disparities in health literacy and oral hygiene behaviors

Education level is a key component of the SDI, and health literacy is strongly linked to oral hygiene practices. In high-SDI countries, public awareness of periodontal disease is generally higher, with preventive measures such as regular dental check-ups and flossing being widely adopted. In contrast, populations in low-SDI regions often lack adequate oral health knowledge, leading to poor plaque control and an increased risk of periodontal disease. Furthermore, high-risk behaviors such as smoking and high-sugar diets, which are more prevalent in low-SDI areas, may further contribute to the onset and progression of periodontal disease (21).

3. Economic accessibility and the influence of healthcare policies

Periodontal treatments (e.g., scaling and root planing, periodontal surgery) are often costly, and healthcare systems in low-SDI countries frequently fail to provide sufficient coverage for such treatments. As a result, patients may delay seeking care due to financial constraints. In comparison, high-SDI countries typically implement more comprehensive public oral health policies, such as universal dental insurance or government-subsidized preventive programs, which help reduce the burden of periodontal disease (19).

The disease burden of periodontitis demonstrates significant variations across age groups and genders. The highest prevalence and case numbers were observed among individuals aged 40–54 years, showing an initial increase followed by a subsequent decline with advancing age. Male patients exhibited slightly higher DALYs and incidence rates compared to females, potentially attributable to factors such as lower prioritization of oral health, higher smoking prevalence, and differential healthcare-seeking behaviors among males (21, 22). These findings suggest that middle-aged/older adults and male populations should be prioritized in periodontitis prevention and treatment strategies.

Projections based on ARIMA and ES models indicate a modest declining trend in global periodontitis incidence, yet the disease burden remains substantial. The ARIMA model predicts an age-standardized incidence rate of 1,854.07 per 100,000 population by 2050, while the ES model yields a more conservative estimate (1,828.73/100,000). Although slight discrepancies exist between the two models' predictions, both consistently demonstrate the persistent burden of periodontitis over coming decades, underscoring the critical need for enhanced global prevention and control measures. Both ARIMA and ES models have been extensively employed in forecasting disease progression patterns (16, 23, 24). This study employs both ARIMA and exponential smoothing (ES) models to predict the future burden of periodontal disease. The selection of these two models is based on their respective strengths and complementary roles in epidemiological forecasting:The ARIMA model effectively captures autocorrelation and trend characteristics in time-series data. The incidence data of periodontal disease exhibit a clear long-term declining trend [with a negative estimated annual percentage change (EAPC) from 1990 to 2021] alongside short-term fluctuations (e.g., a slight rebound after 2010). The differencing component of ARIMA handles non-stationary trends, while the autoregressive (AR) and moving average (MA) terms model serial dependency and random fluctuations, respectively. For a multifactorial chronic disease like periodontal disease, the ARIMA model can flexibly adapt to heterogeneous temporal patterns across different regions through parameter optimization. For instance, when predicting disease burden in high-burden countries such as China, ARIMA better reflects the long-term impacts of demographic shifts.However, the ARMA model (a subset of ARIMA without differencing) has limitations in adapting to long-term trends and incorporating exogenous variables (e.g., policy interventions, population aging), potentially underestimating future shifts in disease burden.

In this study, the ES (exponential smoothing) model serves as a critical complement to our analysis. By assigning greater weight to recent data (via the smoothing coefficient *α*), the ES model demonstrates heightened sensitivity to short-term fluctuations in periodontal disease incidence. This feature is particularly valuable when analyzing the resurgent trend observed post-2010, as the ARIMA model may overemphasize long-term trends and underestimate recent variations.The ES model exhibits strong robustness in predicting periodontal disease-related YLDs (years lived with disability), especially under conditions of high data volatility. However, as a weighted averaging method by nature, the ES model has inherent limitations:Its response to structural breaks (e.g., public health emergencies) may be delayed.It lacks the capacity to incorporate covariates (e.g., smoking rates, oral health policies) into its framework.

The combined use of both models significantly improved prediction reliability. The ARIMA model projected an incidence rate of 1,854.07 per 100,000 by 2050, while the ES model yielded a slightly more conservative estimate of 1,828.73 per 100,000, demonstrating close agreement (a marginal difference of ∼1.4%). This consistency enhances the credibility of the findings.Relying solely on a single model would preclude the assessment of prediction stability. Specifically:ARIMA excels at capturing structured long-term trends, such as the SDI-associated decline in disease burden.ES is more responsive to recent variations, including the impact of sudden public health events.By comparing the prediction intervals of both models (e.g., ARIMA's typically wider 95% CI), a more comprehensive evaluation of future scenario uncertainty can be achieved, thereby providing risk-alert insights for policymaking.

Compared to previous periodontal disease studies based on GBD 2021 data, this study is the first to integrate ARIMA and ES models for long-term burden prediction while revealing their complementary roles. Existing research predominantly relies on single-model approaches (e.g., Joinpoint regression or BAPC models) to analyze periodontal disease trends (25). In contrast, our study innovatively employs ARIMA (to capture long-term structured trends) and ES models (to optimize short-term fluctuation responses) in parallel, with comparative validation enhancing prediction robustness.This dual-model validation strategy represents a novel contribution to periodontal disease forecasting, addressing a critical gap in prior methodologies that risk underestimating the impacts of policy interventions or unexpected disruptions due to single-model limitations.

This study reveals the nonlinear relationship between periodontal disease burden and the Socio-demographic Index (SDI), as well as significant regional heterogeneity. While previous studies predominantly reported a simple negative correlation between SDI and periodontal disease burden (26, 27), our findings demonstrate that:Low-to-middle SDI regions (e.g., Tropical Latin America) exhibit the highest incidence rates, Yet high-SDI countries (e.g., Commonwealth nations) paradoxically bear a heavier DALYs burden, suggesting that economic development does not linearly reduce disease burden—a phenomenon potentially linked to fragmented oral healthcare systems (e.g., declining specialist referral rates) (26). Quantitative analyses using the Slope Index of Inequality (SII) and Concentration Index (CI) further identified diverging trends during 1990–2021: Relative inequalities worsened in high-SDI nations (rising CI),While absolute inequalities improved more substantially in low-SDI regions (declining SII).These findings provide novel empirical support for the equity-oriented targets outlined in WHO's Global Oral Health Action Plan 2025.

This study reveals that while global health inequalities in periodontitis have shown improvement, low-SDI regions continue to bear a disproportionately high disease burden (28). To reduce health inequalities, the following evidence-based interventions are recommended:

1. Strengthen Primary Oral Healthcare Systems

In low-SDI regions, priority should be given to scaling up basic oral health interventions, including:Community water fluoridation programs, School-based oral health education initiative and Periodontal disease screening at primary healthcare facilities.For example, the WHO-endorsed Primary Oral Health Care (POHC) model can enhance preventive coverage by training non-specialist health workers (e.g., community health volunteers) to deliver basic periodontal care (5).

2. Leverage Digital Health Technologies for Preventive Efficiency

Mobile health (mHealth) solutions—such as: Smartphone-based oral health apps, Teledentistry consultations, and AI-assisted periodontal screening tools can significantly improve early diagnosis rates in resource-limited settings. A study conducted in rural India demonstrated that AI-powered image recognition for gingivitis screening substantially increased early detection of periodontal disease (20).

3. Implement Multisectoral Collaborative Interventions

Periodontal disease prevention should extend beyond oral health services and integrate with:Chronic disease management [e.g., combining periodontal screening with diabetes care to mitigate glycemic control deterioration (10)]. Public health policies targeting modifiable risk factors (e.g., tobacco advertising restrictions, promoting reduced refined sugar intake).

Despite employing GBD 2021 data for systematic analysis of periodontal disease trends and utilizing ARIMA and ES models for forecasting, this study has several limitations:

1. Potential Biases in Data Sources

The GBD database relies on nationally reported epidemiological data, which may underrepresent cases in low-SDI countries due to:Incomplete diagnostic and reporting systems (e.g., lack of standardized Community Periodontal Index, CPITN assessments), compromising data comparability.Heterogeneous disease definitions across studies (e.g., inconsistent differentiation between gingivitis and periodontitis), whereas GBD uses uniform ICD codes (K05-K06.9), potentially oversimplifying clinical classifications.

2. Unaccounted Behavioral and Environmental Factors

While smoking and diabetes are documented risk factors for periodontal disease, the GBD dataset only records them as comorbidities, limiting analysis of their dynamic associations with disease trends. Key omissions include:Tobacco control policies, which may reduce future burden but were not quantified.Emerging risks (e.g., refined sugar consumption, air particulate effects on oral mucosa) that could drive unmodeled burden variations.

3. Incomplete Assessment of Health Inequalities

Although the study compared burden disparities across SDI strata, it did not explore:Intra-country disparities (e.g., urban-rural divides, oral health inequities among ethnic minorities).Subpopulations in high-SDI nations (e.g., low-income groups with persistently high periodontal risk), a critical gap given socioeconomic gradients in healthcare access.

## 5 Conclusion

Global Trends and Regional Disparities in Periodontal Disease Burden.While the global burden of periodontal disease demonstrates a declining trend, significant regional disparities persist. The overall decrease in age-standardized incidence rates (ASIR) and disability-adjusted life years (DALYs) masks the disproportionately higher burden observed in low and lower-middle SDI regions compared to high-SDI areas, underscoring the critical influence of socioeconomic determinants on disease prevalence. The distinct patterns of high incidence in Tropical Latin America vs. elevated DALYs in Commonwealth countries suggest the need for region-specific prevention strategies targeting dominant risk factors (e.g., healthcare resource shortages, high smoking prevalence).

The peak prevalence among 40–54-year-olds identifies middle adulthood as a pivotal period for periodontal disease progression, necessitating enhanced early screening and intervention (e.g., routine scaling, periodontal therapy) for this demographic. The higher DALYs observed in male patients likely reflect gender-specific behavioral factors (e.g., lower healthcare utilization, higher tobacco use), warranting tailored health education initiatives for male populations.

Modeling forecasts suggest a modest global incidence decline by 2050, though population aging may attenuate this trend. Passive reliance on epidemiological transitions appears insufficient—proactive measures must include: 1. international collaboration to standardize periodontal surveillance systems, and 2. integration of emerging technologies (e.g., AI-assisted diagnostics) to optimize prevention efficacy.

Future research should integrate multi-omics approaches (particularly microbiomics) and socio-epidemiological methods to elucidate the bidirectional mechanisms linking periodontal disease with systemic conditions. The development of dynamic prediction models incorporating time-varying SDI trajectories and policy intervention variables will enable more precise public health decision-making.

## Data Availability

Publicly available datasets were analyzed in this study. This data can be found here: https://www.healthdata.org/research-analysis/.
